# Influence of Lingual Tonsillar Volume in Patients with Obstructive Sleep Apnea

**DOI:** 10.3390/life12111920

**Published:** 2022-11-18

**Authors:** Yung Jee Kang, Byung Kil Kim, Sang Duk Hong, Yong Gi Jung, Gwanghui Ryu, Hyo Yeol Kim

**Affiliations:** 1 Department of Otorhinolaryngology—Head and Neck Surgery, Samsung Medical Center, Sungkyunkwan University School of Medicine, Seoul 06351, Republic of Korea; 2 Department of Otorhinolaryngology—Head and Neck Surgery, Kyungpook National University of Chilgok Hospital, Hoguk-ro, Buk-gu, Daegu 41404, Republic of Korea

**Keywords:** lingual tonsil volume, polysomnography, obstructive sleep apnea

## Abstract

This study aimed to evaluate the influence of lingual tonsil (LT) volume measured using a three-dimensional (3D) reconstruction volume rendering program on clinical parameters and polysomnography (PSG) results. A total of 100 patients who underwent PSG, computed tomography (CT), and allergy test from April 2016 to April 2020 were randomly selected. LT volume was measured using an imaging software program that enables 3D reconstruction of CT images. PSG parameters were analyzed by dividing the subjects into two groups according to LT volume (each 50 people). Based on the medial volume of 0.863 cm^3^, the upper half LT volume group and the lower half LT volume group were analyzed. Clinical factors such as body weight, neck circumference, body mass index (BMI), and age showed no difference between the two groups. Among PSG parameters, supine arousal index and non-rapid eye movement (NREM) arousal index were significantly higher in the upper half LT volume group (*p* = 0.012, 0.037). However, there was no significant difference in apnea-hypopnea index (AHI) between the upper and lower half LT volume groups (*p* = 0.749). Arousal snoring index and REM arousal index also showed no difference between the two groups. The prevalence of allergic rhinitis was not different in the two groups. High LT volume is associated with NREM arousal and arousal in the supine position, but it is not related to AHI.

## 1. Introduction

The lingual tonsil (LT) is nonencapsulated lymphoid tissue at the posterior tongue base, consisting of Waldeyer’s ring [1,2,3,4]. The LT size varies among people, and LT hypertrophy is thought to contribute to upper airway narrowing, difficult intubation, and dysphagia [5,6,7,8]. Such hypertrophy is also known to cause sleep disordered breathing [7]. Several studies have reported a relationship between LT and body mass index (BMI) or LT and obstructive sleep apnea (OSA), but the results are controversial [4,7,9].

In previous studies, LT hypertrophy is related to laryngopharyngeal reflux (LPR), allergic rhinitis, age, smoking history, and obesity [3,4,7,9,10,11]. As it seems that LT hypertrophy also might be related to OSA, several authors have investigated the relationship; however, most studies failed to prove a relationship between them [4]. These previous studies did not evaluate LT volume directly but as cross-sectional thickness or endoscopic grade [1,4,7,12]. We assumed that an inaccurate grading system for LT hypertrophy is a cause of the unclear association, indicating the need for a more accurate method for measuring LT volume.

In that context, exact estimation of LT volume is needed. We previously reported the association with OSA by measuring parapharyngeal fat volume using AMIRA 5.4 (Mercury Computer Systems/3D Viz group, San Diego, CA, USA) software [13]. This is a validated imaging program that enables three-dimensional (3D) reconstruction of CT imaging and volume measurement. Herein, we directly measured LT volume using CT imaging though AMIRA program and identified influences on clinical parameters and polysomnography (PSG) factors according to LT volume.

## 2. Materials and Methods

### 2.1. Subjects

This study was approved by the Institutional Review Board of our institution (No. 2021-08-050-002). This was a retrospective study performed in a single tertiary institution, Samsung Medical Center. One hundred OSA patients were randomly selected from those who underwent PSG, CT scans, and allergy blood tests from 1 April 2016 to 1 April 2020. The exclusion criteria were patients whose CT scans did not fully cover the lingual tonsil extent, age younger than 18 years, and patients diagnosed with base of tongue cancer or lingual tonsil cancer. All of the patients underwent facial bone CT with a 0.625 mm thin cut. Patient demographics are presented in Table 1.

### 2.2. Evaluation of LT Volume

**Figure 1:**
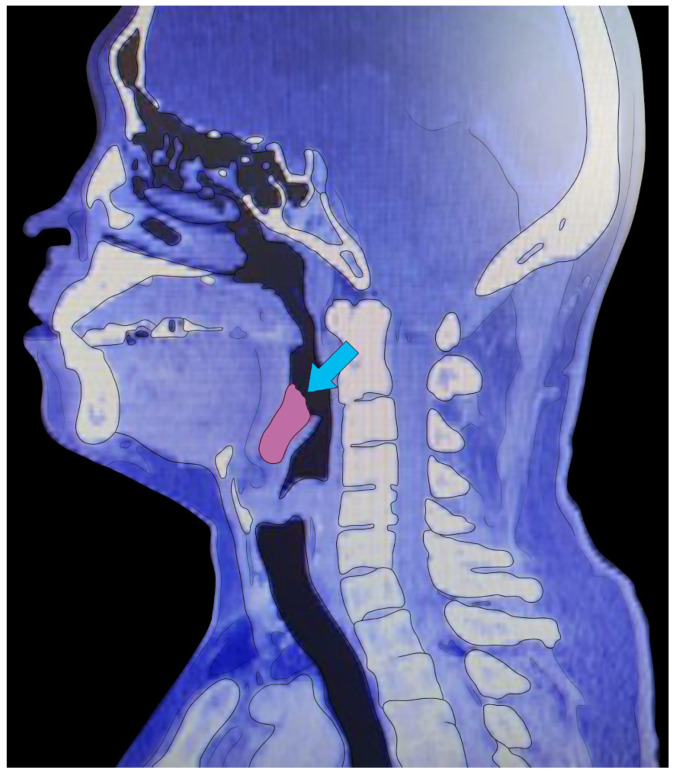
Measurement of lingual tonsil volume using the AMIRA 3D volume rendering program. Arrow indicates the area of lingual tonsil to measure the volume.

LT volume was measured using CT and a validated imaging program, AMIRA 5.4, which enables 3D reconstruction of CT imaging. Axial Digital Imaging and Communications in medicine image files acquired from CT were imported into the AMIRA software program. Sagittal CT images were used to identify the tongue base posterior border, lingual tonsil border, and tonsillar fossa (Figure 1). For measuring LT volume, LT tissue was defined by voxels ranging from Hounsfield unit −200 to +200 in sagittal CT image. In other articles, the average tonsil or lymphoid tissue Hounsfield units is about 50 [14,15]. In AMIRA software program CT sagittal images, we draw a border of LT per cut and define the LT by voxels. The 3D volume calculation was performed through LT voxel summation. As image software allows pixel-level measurements, the error of measurement in the same measuring point is negligible [16].

### 2.3. Polysomnography

An Alice3 (Healthdyne Technologies, Marietta, GA, USA) or Somnologica Studio (Embla Systems, Broomfield, CO, USA) PSG device was used for a one-day overnight diagnostic level 1 PSG exam. During PSG, four-channel electrooculogram, electroencephalogram (EEG; C3/A2, C4/A1, O1/A2, and O2/A1), electromyogram (submental, intercostal, anterior tibialis muscles), and electrocardiogram were used for monitoring [17]. Through piezoelectric bands, abdominal and thoracic movements were assessed. Nasal-oral airflow and oxygen saturation were measured using pulse oximetry [17]. PSG was performed by well-trained technicians and interpreted by one senior doctor, over 15 years of experience. PSG was scored according to the standard rules [18]. The apnea-hypopnea index (AHI) is a main PSG outcome measurement of OSA. The PSG factors of total sleep time, total arousal index, arousal snoring index, spontaneous arousal index, supine arousal index, lateral arousal index, non-rapid eye movement (NREM) arousal index, REM arousal index, AHI, supine AHI, lateral AHI, lowest SaO2 (%), and <90% SaO2 (%) were dependent variables.

### 2.4. Outcome Measures

We classified the enrolled patients into two groups according to the median of LT volume, upper half LT volume group and lower half LT volume group. There are no previous studies address LT volume, there are no reference values to define larger and smaller LT volume. Therefore, we classified two groups, upper half LT volume group and lower half LT volume group according to median value of LT volume. Anthropometric parameters of age, height, weight, BMI, abdominal circumference, hip circumference, lateral neck circumference, and supine neck circumference were dependent variables. Scores on questionnaires of Insomnia Severity Index, Stanford Daytime Sleepiness Index, Epworth Daytime Sleepiness Index, and Reflux Symptom Index (RSI) also were analyzed as dependent variables. The Stanford sleep scale is a 7-point scale reflecting alertness of a patient, and a score of 3 or more represents excessive sleepiness [19]. Epworth Daytime Sleepiness scale contains 8 questions reflecting subjective daytime sleepiness [19]. Each question was scored 0 to 3, and total score higher than 10 points was regarded as excessive sleepiness [19]. The RSI questionnaire includes 9 items about globus, heartburn, breathing, coughing, excessive mucus, and difficulty swallowing [20]. An RSI score higher than 13 points indicates LPR disease [20]. As one previous study suggests that LT hypertrophy was more frequent in allergic rhinitis patients than in those without, we also analyzed LT volume using an ImmunoCAP allergy assay (Phadia AB, Uppsala, Sweden) [3]. Serum total immunoglobulin E (IgE) and specific-IgE in response to house dust mites (*Dermatophagoides Pteronyssinus* and *D. farina)*, cat, tree mixture, weed mixture, dog, and staphylococcal enterotoxin were tested. Allergic and nonallergic group are compared according to LT volume.

### 2.5. Statistical Analysis

All statistical analyses were performed using SAS Version 9.4 statistical software program (SAS Institute, Cary, NC, USA). T-test, Wilcoxon rank sum test, and Chi-Square test were used for analysis of two groups, the upper half LT volume group and lower half LT volume group. Results are represented as mean ± standard deviation or median and interquartile range (IQR) values. Spearman correlation analyses were used to analyze correlation between LT volume and outcome measures. A *p*-value < 0.05 is considered statistically significant.

## 3. Results

Median volume of LT measured using the AMIRA program was 0.863 cm^3^ (IQR, 0.526–1.521 cm^3^). Patients were divided into an upper half group (N = 50) and lower half group of LT volume (N = 50). Range of upper half LT volume groups is from 4.547 cm^3^ to 0.867 cm^3^. Range of lower half LT volume group is from 0.859 cm^3^ to 0.080 cm^3^. First, we compared two groups of LT volume on anthropometric factors. The upper half group of LT volume showed taller height than the lower half group (*p =* 0.041). However, there was no statistically significant difference in terms of age, body weight, BMI, neck circumference, or abdominal circumference between the two groups. Social factors of smoking and alcohol also showed no difference between the two groups. In contrary to a previous result, we did not find any difference of LT volume according to allergic status [3]. These results are presented in Table 2.

Second, we analyzed the influence of LT volume on questions including Insomnia Severity Index, Stanford Daytime Sleepiness Index, Epworth Daytime Sleepiness Index, and RSI. We used subjective questionnaire, RSI on LPR, as symptoms of LPR are meaningful and RSI is a validate tool enough to evaluate LPR [20]. There was no difference between the two groups in terms of RSI. There was no difference in RSI or sleepiness indexes between the two groups (Table 3). The PSG factors including total sleep time, total arousal index, arousal snoring index, spontaneous arousal index, supine arousal index, lateral arousal index, NREM arousal index, REM arousal index, AHI, supine AHI, lateral AHI, lowest SaO2 (%), and <90% SaO2 (%) were analyzed as dependent variables on influence of LT volume (Table 4). In the upper half LT volume group, supine arousal was significantly increased than that of the lower half LT volume group (*p =* 0.012). Lateral arousal index, however, did not show any difference between the two groups (*p* = 0.761). The other parameters of total sleep time, total arousal index, arousal snoring index, spontaneous arousal index, SaO2, and supine AHI showed no statistically significant relationship between the two LT volume groups. While the REM arousal index did not show a difference between the two groups, the NREM arousal index was significantly higher in the upper half LT volume group (*p =* 0.037). AHI also showed no difference between the two groups, indicating that LT volume does not affect the severity of OSA.

Spearman correlation analysis was performed to identify correlation between LT volume and all outcome measures. The result represented that only neck circumference has a weak correlation to LT volume (*r* = 0.200, *p* = 0.049).

## 4. Discussion

As there are no previous studies reporting LT volume in OSA patients, our study uses an innovative method about measuring LT volume through 3D reconstruction volume rendering program.

LT can be identified in a sagittal CT image at the posterior tongue base [14]. As tonsil tissues form a tonsillar crypt, this structure might be helpful to identify LT in imaging. In addition, the border of the LT is the posterior tongue base [14]. Average Hounsfield units of tonsil tissue or lymphoid tissue on non-contrast CT are about 50 [14,15].

Although there is no accurate measurement method for LT, one study suggests a standardized LT grading system using flexible laryngoscopy and CT imaging [21]. They suggest grades 0 to 4 based on LT thickness and lymphoid tissue extent around the posterior tongue base [21].

In the present study, we used the AMIRA software program to measure LT volume through 3D reconstruction using CT images and volume rendering. This is the only technique to measure LT volume directly based on 3D rendering of the LT.

The supine arousal index was increased significantly more in the upper half LT volume group than in the lower half LT volume group. When a patient lies in the supine position, gravity affects the LT and narrows the oropharyngeal airway compared with that in the lateral position [22]. Due to this oropharyngeal airway narrowing, arousal in the supine position shows a significant difference between the two LT volume groups. In the supine position, a patient wakes more frequently. However, neck circumference, body weight, and BMI were not higher in the upper half LT volume group, though they have been reported to have association with arousal in previous research [4]. The upper half LT volume group was taller than the lower half LT volume group, indicating that a tall person has a taller LT and thus greater LT volume. Social factors such as smoking and alcohol showed no difference between the two LT groups. Although one study shows that LT hypertrophy relates to allergic rhinitis, our study indicated that allergic rhinitis is not related to LT volume [3].

In questionnaires, Insomnia Severity Index, Daytime Sleepiness Index, total sleep time, and total arousal index showed no difference between the two LT volume groups. This suggests that larger LT volume does not result in daytime sleepiness or insomnia but does experience greater arousal only in the supine position. In contrast to a previous study showing the relationship between LPR and LT hypertrophy, our study did not show RSI to have a significant relationship with LT volume [11].

As shown in Table 4, there was no significant difference in AHI, supine AHI, or lateral AHI between the two LT volume groups. This demonstrates that LT volume does not affect the severity of OSA. As in previous studies, our analysis revealed LT hypertrophy to not be related with AHI and OSA. From our study, we concluded that LT volume influences supine arousal and NREM arousal rather than AHI, indicating a larger effect on arousal index than AHI. Additionally, lowest SaO2 and <90% SaO2 showed no difference between the two LT volume groups, suggesting that LT volume does not affect O2 saturation.

In the present study, there was significant difference of arousal index during NREM sleep in contrast to REM sleep between the two groups. During NREM sleep, the arousal threshold is higher, indicating that patients are more vulnerable to arousal stimulus than when in REM sleep [23]. Large LT might increase the arousal stimulus more in NREM sleep and be associated with increased arousal during NREM sleep.

Additionally, the NREM arousal index was higher than the REM arousal index. It was shown previously that the arousal index is higher during REM sleep than deep NREM sleep but lower during REM sleep than in light NREM sleep [24]. As OSA patients show a low proportion of deep NREM sleep and a high proportion of light NREM sleep, the NREM arousal index might be higher than the REM arousal index [25]. LT volume is more correlated to arousal index rather than AHI. It emphasizes that LT volume affects to arousal which is new in current knowledge, and it does not seem to be related with OSA severity. Further clinical studies about reduction of LT volume which makes decreased arousal index and sleep efficiency are needed.

Spearman correlation analyses were used to analyze correlation between LT volume and outcome measures. The result represented that only neck circumference has a weak correlation to LT volume (r = 0.200, *p* = 0.049). However, other outcome measures were not significantly correlated with LT volume.

There are some limitations in our study. First, we used data from 100 OSA patients from a single institution. There might be a bias of randomly selection of 100 patients. As we measured LT volume using manually identified borders, some error is likely. Additionally, we only analyzed LT volume between two groups, upper and lower halves of LT volume. No direct relationship between LT volume and clinical factors of PSG were analyzed in our study. Second, we focused on adult LT and not that of children, although different results might be found in children. In our study, we only included adults aged over 18 years old. Although it might seem that pediatric group also has correlation between LT hypertrophy and OSA, CT evaluation was not routinely done in pediatric OSA patients in our institution due to radiation. CT evaluation and LT evaluation through laryngoscopy might be difficult due to cooperation in children. However, further researches are needed for pediatric OSA group. Lastly, although we analyzed only the LT volume measured by CT, a more accurate relationship could be confirmed by applying multimodalities such as magnetic resonance image or drug-induced sleep endoscopy. Future studies are needed on LT volume and should be actively performed to determine its influence on other clinical parameters.

## 5. Conclusions

In summary, arousal in the supine position and NREM arousal index was increased in the upper half LT volume group. LT volume affects NREM sleep more significantly than REM sleep. However, LT volume is not related to AHI and OSA severity.

## Figures and Tables

**Table 1:** Patient Demographics.

	N (Total 100)
Sex	
Male	82
Female	18
Age (years)	
>70	3
60~70	17
50~60	29
40~50	19
30~40	12
20~30	17
10~20	3
BMI (kg/m^2^)	
>40	1
30~40	11
25~30	46
20~25	39
<20	3

BMI = Body Mass Index.

**Table 2:** Influence of LT volume on anthropometric and social factors and allergic rhinitis.

Variable	Statistic	Total	Lower Half LT Volume (*N* = 50)	Upper Half LT Volume (*N* = 50)	*p*-Value
Age	Mean_StdDev	45.13 ± 14.11	46.95 ± 15.78	43.3 ± 12.35	0.420
	Median_IQR	46 [34, 58]	50 [32.5, 61.5]	45 [35, 51]	
Height	Mean_StdDev	170.44 ± 8.84	168.98 ± 7.85	171.9 ± 9.59	0.041 *
	Median_IQR	171 [166, 177]	170 [165, 175]	172.5 [168, 178]	
Weight	Mean_StdDev	76.02 ± 14.19	75.96 ± 16.43	76.08 ± 11.71	0.486
	Median_IQR	75 [67, 85]	73 [66, 85]	76 [70, 85]	
BMI	Mean_StdDev	26.08 ± 3.98	26.48 ± 4.87	25.67 ± 2.83	0.783
	Median_IQR	25.43 [23.58, 27.95]	25.43 [23.46, 28.69]	25.53 [23.99, 27.34]	
Abdominal circumference	Mean_StdDev	89.49 ± 16.05	91.7 ± 10.93	87.28 ± 19.78	0.692
	Median_IQR	91 [84, 97]	90 [85, 97]	92 [84, 96]	
Hip circumference	Mean_StdDev	95.58 ± 15.22	97.88 ± 7.16	93.28 ± 20.14	0.748
	Median_IQR	97 [92.5, 102]	97 [92, 102]	97 [93, 101]	
Lateral neck circumference	Mean_StdDev	37.69 ± 6.08	38.06 ± 3.41	37.31 ± 7.92	0.248
	Median_IQR	38 [36.5, 40]	38 [36, 40]	39 [37, 40]	
Supine neck circumference	Mean_StdDev	38.18 ± 6.15	38.56 ± 3.41	37.8 ± 8.02	0.240
	Median_IQR	38.5 [37, 40.5]	38.5 [36.5, 40.5]	39.5 [37.5, 40.5]	
Smoking	Yes	48	13 (27.7%)	35 (74.5%)	0.815
	No	46	34 (72.3%)	12 (25.5%)	
Alcohol	Yes	61	30 (63.8%)	31 (66.0%)	0.829
	No	33	17 (36.2%)	16 (34.0%)	
Allergic rhinitis	Yes	44	21 (46.7%)	23 (52.3%)	0.674
	No	45	24 (53.3%)	21 (47.7%)	

Bold values indicate significant *p*-values. * *p*-value < 0.05 significant. LT = Lingual Tonsil, StdDev = Standard Deviation, IQR = Interquartile Range.

**Table 3:** Influence of LT volume on questionnaire items.

Variable	Statistic	Total	Lower Half LT Volume (*N* = 50)	Upper Half LT Volume (*N* = 50)	*p*-Value
Insomnia index	Mean_StdDev	9.06 ± 5.9	9.24 ± 5.95	8.9 ± 5.91	0.733
	Median_IQR	9 [4, 14]	10 [4, 14]	8 [4.5, 13]	
Stanford daytime sleepiness index	Mean_StdDev	2.91 ± 1.06	3.1 ± 1.12	2.71 ± 0.96	0.070
	Median_IQR	3 [2, 3]	3 [2, 3]	3 [2, 3]	
Epworth daytime sleepiness index	Mean_StdDev	10.9 ± 5	11.14 ± 4.31	10.65 ± 5.65	0.631
	Median_IQR	10.5 [8, 14]	12 [8, 14]	10 [6, 14]	
RSI score	Mean_StdDev	11.06 ± 8.6	9.85 ± 8.5	12.21 ± 8.63	0.158
	Median_IQR	9 [4, 16.5]	7 [3, 12]	10 [5, 18]	

*p*-value < 0.05 significant. LT = Lingual Tonsil, StdDev = Standard Deviation, IQR = Interquartile Range.

**Table 4:** Influence of LT volume on PSG factors.

Variable	Statistic	Total	Lower Half LT Volume (*N* = 50)	Upper Half LT Volume (*N* = 50)	*p*-Value
AHI	Mean_StdDev	32.69 ± 22.34	32.15 ± 23.09	33.24 ± 21.78	0.749
	Median_IQR	27.2 [17.15, 43.8]	25.95 [15.9, 44]	28.7 [17.2, 43.6]	
Supine AHI	Mean_StdDev	35.3 [19.95, 66.7]	34.7 [19.3, 47.5]	39.9 [20.3, 68.1]	0.298
	Median_IQR	0~104.1	0.3~104.1	0~94.9	
Lateral AHI	Mean_StdDev	17.91 ± 28.03	17.37 ± 20.73	18.43 ± 33.81	0.938
	Median_IQR	7.65 [2, 22.9]	8.9 [1.4, 21.9]	7.2 [2.4, 23.9]	
Lowest SaO2 (%)	Mean_StdDev	82.16 ± 8.05	82.48 ± 7.26	81.84 ± 8.83	0.978
	Median_IQR	83.5 [79, 88]	84 [79, 88]	83 [80, 88]	
<90% SaO2 (%)	Mean_StdDev	4.45 ± 8.96	3.71 ± 7.51	5.18 ± 10.24	0.652
	Median_IQR	0.5 [0.1, 3.55]	0.3 [0.1, 3.2]	0.7 [0, 3.6]	
Total sleep time	Mean_StdDev	370.3 ± 57.07	373.49 ± 59.98	367.11 ± 54.43	0.579
	Median_IQR	370.25 [334.5, 408.75]	373.25 [330, 407.5]	366 [343, 416]	
Total arousal index	Mean_StdDev	176.45 ± 208.92	182.64 ± 286.56	170.26 ± 77.39	0.076
	Median_IQR	147 [109, 194]	137 [90, 192]	161 [127, 205]	
Arousal snoring index	Mean_StdDev	0.6 ± 0.96	0.69 ± 1.18	0.5 ± 0.68	0.733
	Median_IQR	0.2 [0, 0.75]	0.2 [0, 0.7]	0.2 [0, 1]	
Spontaneous arousal index	Mean_StdDev	3.57 ± 4.34	3.47 ± 4.89	3.66 ± 3.76	0.493
	Median_IQR	2.5 [0.95, 4.55]	2.6 [0.9, 4.3]	2.3 [1, 4.9]	
Supine arousal index	Mean_StdDev	33.39 ± 18.93	29.12 ± 17.84	37.65 ± 19.19	0.012 *
	Median_IQR	28.75 [18.55, 43.4]	24.15 [15, 39.1]	31.85 [23.5, 54.5]	
Lateral arousal index	Mean_StdDev	18.46 ± 24.43	17.24 ± 14.29	19.6 ± 31.22	0.761
	Median_IQR	13.8 [7.1, 22.4]	14.7 [6, 25.8]	13.15 [7.5, 20]	
REM arousal index	Mean_StdDev	21.77 ± 15.73	22.09 ± 15.38	21.45 ± 16.23	0.741
	Median_IQR	18.55 [9.5, 28.5]	19.1 [10.6, 30.8]	17.9 [9, 27.4]	
NREM arousal index	Mean_StdDev	27.27 ± 14.14	24.7 ± 13.94	29.85 ± 14	0.037 *
	Median_IQR	25.05 [16.85, 34.35]	22.95 [13.8, 34.3]	27.6 [20.8, 35]	

Bold values indicate significant *p*-values. * *p*-value < 0.05 significant. LT = Lingual Tonsil, PSG = Polysomnography, AHI = Apnea Hypopnea Index, SaO2 = Arterial O2 Saturation, REM = Rapid Eye Movement, NREM = Non Rapid Eye Movement, StdDev = Standard Deviation, IQR = Interquartile Range.

## Data Availability

The data presented in this study are available within the article.
